# Editorial: Online Social Communication: Establishing, Maintaining, and Ending Online Relationships

**DOI:** 10.3389/fpsyg.2022.841620

**Published:** 2022-02-07

**Authors:** Graham G. Scott, Gordon P. D. Ingram, Christopher J. Hand

**Affiliations:** ^1^School of Education and Social Sciences, University of the West of Scotland, Paisley, United Kingdom; ^2^Departamento de Psicologia, University de Los Andes, Bogota, Colombia; ^3^School of Education, University of Glasgow, Glasgow, United Kingdom

**Keywords:** online relationships, communication, social media, online self-presentation, online impression formation

Online communication has evolved rapidly over the past two decades, especially since the implementation of web 2.0 technology. As social media and direct messaging platforms become more widespread, and technology becomes more mobile, the way we communicate with friends, family, colleagues, and romantic partners becomes more diverse. The distinction between the on- and off-line worlds is more ambiguous. Relationships are now not solely on- or off-line, but technology allows us to communicate with members of our social network using a selection of digital tools. With an increasing range of communication platforms offering different affordances, it is important to understand how users utilize the communication mediums available to them, and what impact this can have not only on their relationships, but on them as individuals. To further explore this topic in more detail we launched our Research Topic *Online Social Communication: Establishing, maintaining, and ending online relationships*.

We received nine insightful manuscripts from 31 authors in nine countries covering the distinct specialized sections of Human-Media Interaction and Personality and Social Psychology. The research falls into three areas: (1) online self-presentation, including false self-presentation; (2) online impression formation based on online communications; and (3) online communication and wellbeing.

A first line of research focuses on how users self-present online. Bong and Kim investigated motivations for deceptive self-presentation on Instagram, and the mental and behavioral outcomes associated with it. They demonstrated that users who scored highly in “need for approval” reported lying more often when self-presenting online, and that such behavior increased depression, perceived popularity, and deleting behaviors. While deceptive self-presentation increased depression, perceived popularity acted as a buffer against depression. Zhang et al. examined online self-presentation by looking into the option users have to hide or delete content they post on WeChat after a designated amount of time. Users who utilized the time-limit settings were found to post more frequently, use privacy settings more often, and had smaller audiences. Users who both utilized the settings and scored higher on measures of life changes, self-monitoring, posting frequency, and audience size, but lower on perceived stress were more likely to select a relatively short time limit for the content they posted. Finally, in a review of online identity reconstruction, Huang et al. summarized the theoretical and methodological approaches to research around online identity reconstruction, including examining the predictors and effects of the phenomenon, and provided an overview of the thematic patterns of recent research.

A second line of research takes a different perspective, investigating how we form impressions of others online. Qin et al. investigated how online self-disclosure influenced first impression formation. Specifically, they examined how the valence of self-disclosure (mostly negative, balanced, mostly positive) in WeChat profiles influenced first impressions of an unknown potential collaborator. Their findings showed that dominantly positive self-disclosures were associated with greatest likability; predominantly negative self-disclosures were associated with the lowest levels of likability. Perceived trustworthiness mediated the effect of self-disclosure type on likability. Kong et al. examined the relationship between styles of social media use and vulnerable narcissism. They demonstrated that active and passive use of social media platforms are linked to upwards and downwards social comparison, and that both types of use indirectly predict vulnerable narcissism. Sullivan's study explored the link between attachment jealousy and online jealousy in response to ambiguous hypothetical online scenarios. Participants were all in relationships, and it was found that the link between attachment anxiety and jealousy increased as participants' attitudes to online communication became more negative.

A third line of research looks at how individuals interact with social media and what impact this has on their wellbeing. Ostic et al. recruited 940 social media users from Mexico, gathering data on participants' social media use, social capital, social isolation, smartphone use, phubbing, and psychological wellbeing. Through Structural Equation Modeling, they found that there was an overall positive (but indirect) effect of social media usage on psychological wellbeing—mainly due to effects of bonding and bridging social capital. In a mixed-methods study, He and Liu used semi-structured interviews to identify eight factors that could produce social media fatigue in young people. In a subsequent regression analysis of the results of an online questionnaire, they found that negative social comparison, dysfunctional interactions, informational and social overload, impression management (the most important factor in the model), and poor intergenerational communication were all significant predictors of social media fatigue. Self-efficacy turned out to be a positive factor, rather than a negative one as predicted, while privacy anxiety had no effect. Negative social comparisons were also studied by Lim et al. Noting that studies of the relationship between social comparison and self-esteem have typically shown only weak and unreliable effects, they proposed a novel, “evolutionary mismatch” hypothesis to account for this. According to this hypothesis, negative social comparison with the unrealistic, idealized depictions of other people's lives portrayed on social media only impacts self-esteem when an individual's social network is small enough to correspond to the sort of size it would have had in our evolutionary past. They found a relationship between social media use and low self-esteem in people with social networks of around 150 connections. Yet with many of their participants reporting networks numbering over 1,000 online “friends,” it seems that in these individuals social comparison was not perceived as taking place with real social connections, and thus social media use did not adversely affect self-esteem.

Taken together these findings highlight how different ways of interacting with social media and online communication platforms can impact on users. Both actively self-presenting online, and passively consuming content, can lead to wellbeing-related outcomes. As a limitation we highlight the fact that most studies in the Research Topic utilize cross sectional designs, and thus it is difficult to establish any truly causal relationships between the variables investigated. However, we are confident the studies selected for this Research Topic offer valuable contributions to the area of online communication and offer an insight into the impact interactions with such technologies can have on individual users.

## Author Contributions

GI and CH summarized two articles each and proof read the final version of the article. GS summarized the remaining articles and wrote the editorial. All authors contributed to the article and approved the submitted version.

## Conflict of Interest

The authors declare that the research was conducted in the absence of any commercial or financial relationships that could be construed as a potential conflict of interest.

## Publisher's Note

All claims expressed in this article are solely those of the authors and do not necessarily represent those of their affiliated organizations, or those of the publisher, the editors and the reviewers. Any product that may be evaluated in this article, or claim that may be made by its manufacturer, is not guaranteed or endorsed by the publisher.

